# Supplementary material for: Resolving the full spectrum of human genome variation using Linked-Reads
Source: Genome Res. 2019 Apr;29(4):635–45. doi: 10.1101/gr.234443.118 (PMC6442396; doi:10.1101/gr.234443.118)
Supplement: Supplemental Material [file supp_gr.234443.118_Supplemental_longranger-2.2.zip › longranger-2.2.tar.bz2!/lr2.2-public/lib/stan/cmdstan/stan/doc/api/html/to__var_8hpp_source.html]

Stan: src/stan/agrad/rev/matrix/to\_var.hpp Source File


|  |  |
| --- | --- |
|  | Stan  2.5.0  probability, sampling & optimization |


- Main Page
- Related Pages
- Namespaces
- Classes
- Files

- File List
- File Members

All Classes Namespaces Files Functions Variables Typedefs Enumerations Enumerator Friends Macros Pages

- src
- stan
- agrad
- rev
- matrix

to\_var.hpp

Go to the documentation of this file.

1 #ifndef STAN\_\_AGRAD\_\_REV\_\_MATRIX\_\_TO\_VAR\_HPP

2 #define STAN\_\_AGRAD\_\_REV\_\_MATRIX\_\_TO\_VAR\_HPP

3

4 #include <vector>

5 #include <stan/math/matrix/Eigen.hpp>

6 #include <stan/math/matrix/typedefs.hpp>

7 #include <stan/agrad/rev/var.hpp>

8 #include <stan/agrad/rev/matrix/typedefs.hpp>

9

10 namespace stan {

11  namespace agrad {

12

21  inline var to\_var(const double& x) {

22  return var(x);

23  }

32  inline var to\_var(const var& x) {

33  return x;

34  }

43  inline matrix\_v to\_var(const stan::math::matrix\_d& m) {

44  matrix\_v m\_v(m.rows(), m.cols());

45  for (int j = 0; j < m.cols(); ++j)

46  for (int i = 0; i < m.rows(); ++i)

47  m\_v(i,j) = m(i,j);

48  return m\_v;

49  }

58  inline matrix\_v to\_var(const matrix\_v& m) {

59  return m;

60  }

70  inline vector\_v to\_var(const stan::math::vector\_d& v) {

71  vector\_v v\_v(v.size());

72  for (int i = 0; i < v.size(); ++i)

73  v\_v[i] = v[i];

74  return v\_v;

75  }

85  inline vector\_v to\_var(const vector\_v& v) {

86  return v;

87  }

97  inline row\_vector\_v to\_var(const stan::math::row\_vector\_d& rv) {

98  row\_vector\_v rv\_v(rv.size());

99  for (int i = 0; i < rv.size(); ++i)

100  rv\_v[i] = rv[i];

101  return rv\_v;

102  }

112  inline row\_vector\_v to\_var(const row\_vector\_v& rv) {

113  return rv;

114  }

115

116  }

117 }

118 #endif

typedefs.hpp

stan::agrad::to\_var

var to\_var(const double &x)

Converts argument to an automatic differentiation variable.

**Definition:** to\_var.hpp:21

typedefs.hpp

stan::math::matrix\_d

Eigen::Matrix< double, Eigen::Dynamic, Eigen::Dynamic > matrix\_d

Type for matrix of double values.

**Definition:** typedefs.hpp:23

stan::agrad::matrix\_v

Eigen::Matrix< var, Eigen::Dynamic, Eigen::Dynamic > matrix\_v

The type of a matrix holding stan::agrad::var values.

**Definition:** typedefs.hpp:21

stan::agrad::vector\_v

Eigen::Matrix< var, Eigen::Dynamic, 1 > vector\_v

The type of a (column) vector holding stan::agrad::var values.

**Definition:** typedefs.hpp:29

var.hpp

stan::agrad::row\_vector\_v

Eigen::Matrix< var, 1, Eigen::Dynamic > row\_vector\_v

The type of a row vector holding stan::agrad::var values.

**Definition:** typedefs.hpp:37

Eigen.hpp

stan::agrad::var

Independent (input) and dependent (output) variables for gradients.

**Definition:** var.hpp:27

stan::math::vector\_d

Eigen::Matrix< double, Eigen::Dynamic, 1 > vector\_d

Type for (column) vector of double values.

**Definition:** typedefs.hpp:30

stan::math::row\_vector\_d

Eigen::Matrix< double, 1, Eigen::Dynamic > row\_vector\_d

Type for (row) vector of double values.

**Definition:** typedefs.hpp:37


---

[ Stan Home Page ]

*© 2011–2014,
Stan Development Team.*
